# Synthesis of High Specific Surface Lithium-Ion Sieve Templated by Bacterial Cellulose for Selective Adsorption of Li^+^

**DOI:** 10.3390/molecules28073191

**Published:** 2023-04-03

**Authors:** Xi Zhang, Xudong Zheng, Tongtong Xu, Yuzhe Zhang, Guomeng Li, Zhongyu Li

**Affiliations:** School of Environmental Science and Engineering, Changzhou University, Changzhou 213164, China

**Keywords:** lithium ion sieve, bacterial cellulose, hydrothermal method, titanium, selective adsorption

## Abstract

In recent years, with the development of batteries, ceramics, glass and other industries, the demand for lithium has increased rapidly. Due to the rich lithium resources in seawater and salt-lake brine, the question of how to selectively adsorb and separate lithium ions from such brine has attracted the attention and research of many scholars. The Li-ion sieve stands out from other methods thanks to its excellent special adsorption and separation performance. In this paper, mesoporous titanium dioxide and lithium hydroxide were prepared by hydrothermal reaction using bacterial cellulose as a biological template. After calcination at 600 °C, spinel lithium titanium oxide Li_2_TiO_3_ was formed. The precursor was eluted with HCl eluent to obtain H_2_TiO_3_. The lithium titanate were characterized by IR, SEM and X-ray diffraction. The adsorption properties of H_2_TiO_3_ were studied by adsorption pH, adsorption kinetics, adsorption isotherm and competitive adsorption. The results show that H_2_TiO_3_ has a single-layer chemical adsorption process, and has a good adsorption effect on lithium ions at pH 11.0, with a maximum adsorption capacity of 35.45 mg g^−1^. The lithium-ion sieve can selectively adsorb Li^+^, and its partition coefficient is 2242.548 mL g^−1^. It can be predicted that the lithium-ion sieve prepared by biological template will have broad application prospects.

## 1. Introduction

With the increasing demand for batteries, the demand for metals such as lithium, cobalt and nickel will also increase, which means that the number of mining plans will continue to grow. For environmental ecology, this is not a good thing. Extracting lithium will directly affect water resources and environments. At present, many scientists focus on recycling waste lithium batteries and reusing existing elements, so as to reduce the damage to the overall environment. At the same time, some scientists are exploring another lithium mining method, such as extracting lithium from seawater.

Lithium is the rarest element among alkali metals [1]. The lightest metal, it is a silver-white metal with strong chemical activity, and can synthesize inorganic and organic compounds of lithium with various elements. Lithium and a variety of metals constitute light alloys, wear-resistant alloys, etc. Al-Li and Mg-Li alloys will become new structural materials for the next generation of aerospace industry. Lithium resources in China are mainly distributed in the Qinghai-Tibet Plateau, Xinjiang, Inner Mongolia, Sichuan and Jiangxi, accounting for 25.6% of the world’s lithium reserves, ranking second in the world [2]. Among them, salt-lake brine lithium resources are the main form of lithium resources in China, accounting for about 85% of the total proved lithium reserves in China [3]. Therefore, it is very important to study how to transform lithium extraction technology and to extract lithium efficiently from salt-lake brine [4]. At present, there are many known methods for the adsorption and separation of Li^+^ from salt-lake brine. For example, the precipitation method [5], electrochemical method [6], solvent extraction method [7,8], ion exchange method [9,10], and so on. Among these, the ion-exchange method stands out from the many lithium extraction methods because of its simple process, high recovery rate and economic and green advantages [11]. Some adsorbents are in powder form and have poor fluidity and permeability. In the process of elution and regeneration, the corrosion damage rate of the ion sieve adsorbent is large. These disadvantages limit the current application of lithium adsorbents. Therefore, at the core of the lithium adsorption method, it is particularly important to find excellent adsorption materials with high selectivity to Li^+^ and good stability including thermal stability and mechanical stability.

Compared with some natural inorganic minerals and carbon materials [12], there is a lack of adsorption selectivity for Li^+^ [13]. The lithium ion sieve has the characteristics of selective adsorption of Li^+^ because it is first inserted into the precursor by Li^+^ and then eluted by eluent [14]. As the main representative of lithium ion sieves, spinel manganese oxide ion sieves have high specific adsorption for Li^+^ after acid elution of precursors. For example, Gao [15] synthesized representative adsorbent Li_1.6_Mn_1.6_O_4_ for lithium ion sieves. Its maximum adsorption capacity can reach 44 mg g^−1^. Similarly, Yang [16] made a series of research into LiOH, Mn(CH_3_COO)_2_, H_2_O_2_ and ethanol, using the sol gel method, hydrothermal method and low-temperature solid-phase method combined with manganese oxide lithium ion sieve [17]. Keik [18] synthesized a lithium-ion sieve with microtubule morphology using manganese-oxidizing fungus as a biological template by calcination solid-phase bonding. The effect of adsorption capacities at different calcination temperatures was studied. Structural and morphological characterization and adsorption experiments show that the weight fraction of the spinel lithium manganese oxide ion sieve also changes with different calcination temperatures, and this does affect its crystallinity, thus the choice of calcination temperature directly affects the adsorption capacity of the lithium ion sieve [19]. In Song et al.’s work, spinel structure Li_1+x_ Mn_2-x_O_4_ materials for lithium ion-sieve precursor were synthesized by a high temperature solid state method. Their results show that the Li_1.3_Mn_1.7_O_4_ material had the largest adsorption capacity and it reached up to 24.06 mg g^−1^ when the pH value was 12 and the adsorption time was 10 h [20]. However, the current Li-Mn-O ion sieves have the same shortcomings in the process of acid pickling: the appearance of Mn^2+^ in the elution process causes partial dissolution of the ion sieves, which reduces the repeatability of the lithium-ion sieves. On the other hand, the appearance of titanium-based lithium-ion sieves compensated for the dissolution of Ti during acid pickling. For example, Shuler Wang, Ping Li and others synthesized β-Li_2_TiO_3_ by a hydrothermal method from TiO_2_ and LiOH·H_2_O [21]. Compared with manganese ion sieves, the dissolution rate of Ti is reduced, while Li^+^ is highly selectively adsorbed by manganese ion sieves. Cheng-Long Yua, Kazumi chi Yanagisawa [22], synthesized pure Li_2_TiO_3_ nanoparticles by hydrothermal reaction of anatase TiO_2_ and LiOH·H_2_O, and studied the formation of Li_2_TiO_3_, the diffusion and insertion mechanism of lithium ions [23]. In Ramesh et al.’s work, anatase type TiO_2_ and Li_2_CO_3_ were mixed, ground and heated in an alumina crucible at 700 °C in air to obtain the lithium-ion sieve precursor (Li_2_CO_3_). The measured adsorption capacity of the adsorbent is 32.6 mg g^−1^ [24]. Unfortunately, most of the known lithium-ion sieves are powdered, which is not conducive to the mass production of ion sieves in practical applications. Therefore, the preparation of mesoporous nanomaterials to synthesize the lithium ion sieve is of special significance [25]. Bacterial cellulose (BC) is a polymer metabolized by microorganisms. BC is considered a potential water treatment material because of its unique three-site network structure, highly specific surface area, low environmental cost and high porosity. Cellulose is a polysaccharide with a wide coverage and is highly abundant in nature [26]. High abundance and low price mean that it has naturally attracted the attention of scientific researchers in recent years. However, natural cellulose contains some impurities such as lignin and hemicellulose, and has a coarse fiber diameter which especially affects the performance of cellulose [27]. Bacterial cellulose (BC) is synthesized by microorganisms under different conditions, and the chemical structure of BC is the same as that of plant cellulose [28]. The difference is that BC has the advantages of higher purity and finer fiber diameter than plant cellulose. Due to the formation of ultrafine network structures of bacterial cellulose and the “nano-effect”, bacterial cellulose has the characteristics of high water absorption and water retention, high permeability to liquids and gases, high wet strength, and in-situ processing and molding, especially in wet state. With high purity and excellent performance it can be widely used in many fields.

In this study, we aimed to prepare titanium dioxide mesoporous membrane with BC as a template, and then reacted with LiOH to get Li_2_TiO_3_. The precursor is stabilized by high temperature sintering, and then the mesoporous potassium ion sieve is obtained after acid elution. The adsorption effect and mechanism of this ion sieve with BC as a template were studied by adsorption experiments. It was characterized by scanning electron microscope (SEM), Fourier transform infrared spectroscopy (FT-IR), X-ray photoelectron spectroscopy (XPS), BET and thermogravimetric analysis (TGA). Through a series of static adsorption experiments, the adsorption behaviors such as pH value, adsorption kinetics, adsorption isotherm, adsorption thermodynamics, selectivity and regeneration were studied. See the results and discussion section for details.

## 2. Results and Discussion

### Characterizations of Li_2_TiO_3_ and H_2_TiO_3_

**Figure 1:**
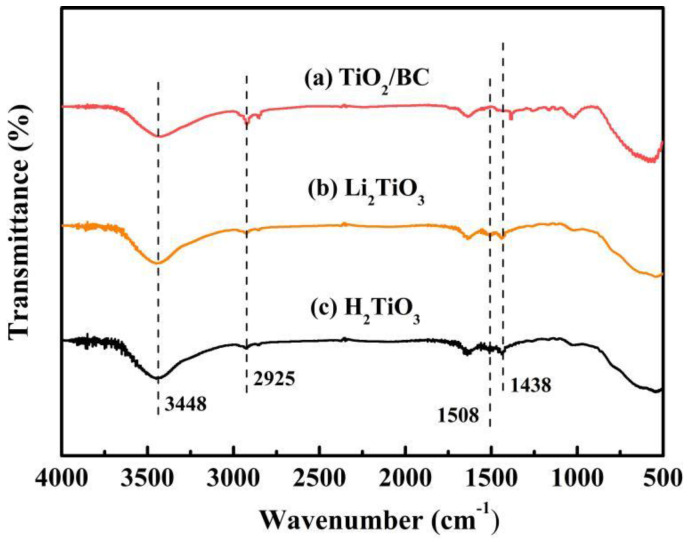
(**a**) FT-IR spectra of TiO_2_/BC; (**b**) Li_2_TiO_3;_ (**c**) H_2_TiO_3_.

**Figure 2:**
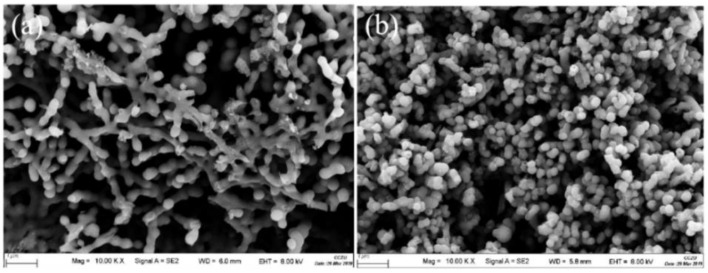
(**a**) SEM image of TiO_2_/BC; (**b**) SEM images of Li_2_TiO_3_.

**Figure 3:**
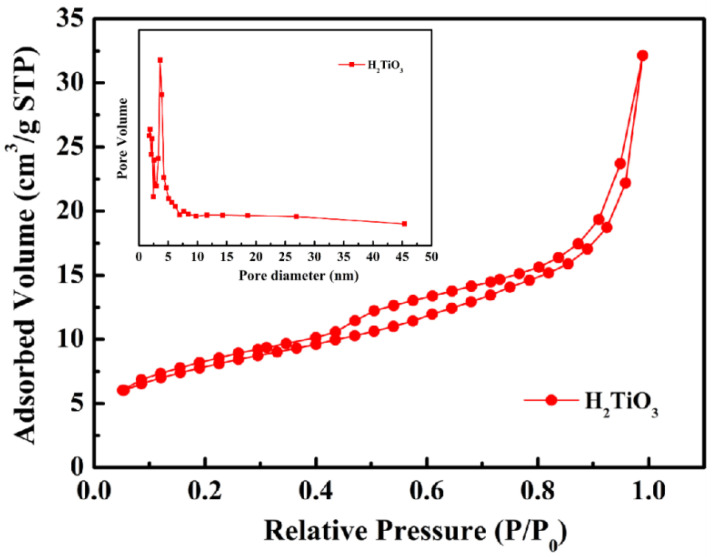
Nitrogen adsorption–desorption isotherm of H_2_TiO_3_. Inset showing pore size distributions.

In order to analyze the functional groups of TiO_2_, Li_2_TiO_3_ and H_2_TiO_3_, FT-IR analysis was performed and presented in Figure 1. Broad peaks at 3448 and 2925 cm^−1^ were observed for three materials’ FT-IR spectra, which can be interpreted as O-H and C-H stretching vibrations, respectively. Among them, OH peak was observed. This peak was due to the isolated O-H bond not participating in the interaction with other hydroxyl groups. In addition, compared with the FT-IR spectra of TiO_2_, the new peaks at 1438 and 1508 cm^−1^ appeared after lithium insertion, which belonged to the characteristic’s vibration of the Li-O-Ti band. This indicates that the precursor (Li_2_TiO_3_) was formed. In the case of Li_2_TiO_3_, the disappearance of peaks at 1438 and 1508 cm^−1^ shows successful elution of lithium ions, and that the preparation of the lithium-ion sieve (H_2_TiO_3_) was completed. As can be seen in the SEM diagram in Figure 2a, the titania films formed with bacterial cellulose as template feature a pronounced high pore volume network structure. After the hydrothermal calcination with LiOH, the films become denser with crystal growth and aggregate, resulting in the formation of spinel Li_2_TiO_3_ (Figure 2b). The nitrogen sorption data of H_2_TiO_3_ showed a BET surface area of 27.4006 m^2^/g. Based on the adsorption desorption isotherm of nitrogen, Figure 3 shows that the pore structure in the material is consistent with the results of SEM. It can further be inferred that bacterial cellulose as template is effective.

**Figure 4:**
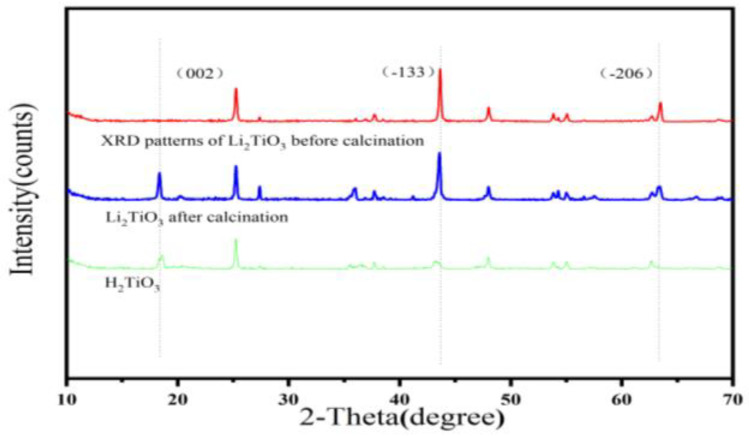
XRD mode of Li_2_TiO_3_, calcined Li_2_TiO_3_ and H_2_TiO_3_.

As shown in Figure 4, the XRD pattern of the Li_2_TiO_3_ before calcination, Li_2_TiO_3_ after calcination and H_2_TiO_3_ were collected at the 2θ angle from 10 to 70°. In the XRD pattern of the Li_2_TiO_3_, a new diffraction peak at (002) appeared after calcination, which was due to the growth of the (002) diffraction peak needing a higher calcination temperature [29]. The occurrence of diffraction peaks of (002), (−133), (−206) and (062) can be seen in Figure 4, indicating that Li^+^ is sequentially inserted into TiO_2_, which matched well with the pure monoclinic crystal of Li_2_TiO_3_ [30]. After eluting, the position of the diffraction peaks can be observed to be about the same, and the diffraction peak of (−133) and (−206) almost disappears, which proves that an exchange occurs in Li^+^/H^+^ and the formation of H_2_TiO_3_ structure.

## 3. Experiment

### 3.1. Sample Materials and Reagents

CS was supplied by Sean Chemical Technology CO., Ltd. (Shanghai, China) with a degree of deacetylation of more than 95%. The molecular equation of CS is (C_6_H_11_NO_4_)n and the molecular weight of the CS unit is about 161.2. 2H12C4 was purchased from TCI Chemical Industry Development CO., Ltd. (Shanghai, China). ECH was purchased from Shanghai Ling Feng Chemical Reagent CO., Ltd. Sodium hydride (NaOH) (60% dispersion in mineral oil), potassium bromide and lithium chloride (anhydrous grade, 98%) were supplied by Aladdin Reagent CO., Ltd. (Shanghai, China). Acetic acid was offered by Shanghai Shen bob Chemical CO., Ltd located in Baoshan District, Shanghai. Ethanol, hydrochloric acid and sodium hydroxide were acquired from Sinopharm Chemical Reagent CO., Ltd located in Baoshan District, Shanghai. All reagents were analytically pure and were used without further purification.

### 3.2. Instruments

The morphology and microstructure of the synthesized adsorbent were observed by a SUPRA scanning electron microscope (SEM; Carl Zeiss, Oberkochen, Germany). Infrared spectra (4000–400 cm^−1^) was recorded on a Nicolet iS5 Fourier-transform infrared spectrometer (FT-IR; Nicolet, Madison, WI, USA). X-ray photoelectron spectroscopy (XPS) was used to surface the chemical characterization of the materials (Thermo ESCALAB 250XI). Nitrogen adsorption–desorption isotherms and the specific surface area were tested by an ASAP2460 Physical Adsorber (Micromeritrics, Norcross, GA, USA). Inductively coupled plasma optical emission spectrometer (ICP-OES, VISTA-MPX) was applied to measure the metal ion concentration. The thermal stability of the sample was determined by a Labsys Evo STA thermogravimetric analyzer (Seta ram Instrumentation, Caloire, France).

### 3.3. Static Adsorption Experiment

In the adsorption experiment, H_2_TiO_3_ was used as an adsorbent to adsorb lithium ions. The effects of pH adsorption time, reaction temperature and initial lithium-ion concentration on the adsorption performance of H_2_TiO_3_ were systematically studied. The lithium-ion sieve H_2_TiO_3_ was placed in LiCl solution with a certain concentration. After adsorption, the solution was separated from the adsorbent by centrifugation at 10,000 rpm min^−1^ for minutes, and then the residual Li^+^ concentration in the solution was determined by ICP-OES. The experiment was completed at 25 °C.

Effect of solution pH: 10 mg lithium-ion sieve H_2_TiO_3_ was immersed into 10 mL Li^+^ reserve solution (1 gL^−1^) and the pH adjusted to 4.0~11.0 by using 0.05~0.5(M) HCI and NaOH solution. The experimental temperature was 25 °C. When the adsorption equilibrium was reached, the final concentration of Li^+^ was determined by ICP-OES.

Kinetic experiment: At a pH of 1.0 and a constant temperature of 25 °C, 10 mg of adsorbent lithium-ion sieve H_2_TiO_3_ was put into 10 mL of Li stock solution (1 gL^−1^) for adsorption, then quickly sampled and filtered according to the set time interval to test the dynamic data. The residual Li^+^ concentration after adsorption at different intervals was determined by ICP-OES.

Isothermal experiment: The experiment was carried out at a of pH 1.0 and a constant temperature control at 25 °C. 10 mg of adsorbent NTO-NCC was placed in a series of concentrations of 10 mL of stock solution (100 mg^−2^gL^−1^). The equilibrium concentration of residual Li^+^ after adsorption was determined by ICP-OES.

Selectivity experiment: The main metal ions K^+^, Ca^2+^, Na^+^, Mg^2+^ and Li^+^ in salt lake water were selected by simulation to prepare the mixed solution. The concentration of each ion was 1 gL^−1^, and the competitive adsorption experiment was carried out at pH 11.0 and a temperature of 25 °C. Finally, the concentration of each ion in the residual solution was determined by ICP-OES.

### 3.4. Synthesis of the Li_2_TiO_3_

Bacterial cellulose (BC) hydrogel, titanium ethoxide (TEOT), lithium hydroxide (LiOH). Deionized water was used in all experiments. Hydrochloric acid (HCl) and other drugs used are analytically pure, without the need for further processing and purification. Bacterial cellulose (BC) hydrogel was placed in deionized water for 15 min to achieve the swelling effect. After freezing with liquid nitrogen and freeze-drying, bacterial cellulose (BC) aerogel with a network structure was obtained. The bacterial cellulose (BC) aerogel was immersed in titanium ethoxide (TEOT) solution for 2 h. Then, after alternately rinsing with ethanol and ultrapure water four to five times, it was placed in ultrapure water and mechanically stirred for 2 h, and the product was repeatedly washed with deionized water. Finally, the bacterial cellulose film material TiO_2_/BC wrapped in TiO_2_ was obtained after drying in the oven (Huang et al. 2016). TiO_2_ with a weight of 0.826 g was obtained by calcining at 600 °C for 6 h at 4 °C/min in a tubular furnace. The TiO_2_ was dissolved in 10.45 mL of H_2_O with 0.5 g of LiOH. With a Li:Ti molar ratio of 2:1, unstable spinel Li_2_TiO_3_ is obtained after hydrothermal reaction at 180 °C for 18 h. The stable spinel Li_2_TiO_3_ was obtained after being calcined at 4 °C/min at 600 °C for 6 h in a tube furnace.

### 3.5. Synthesis of the H_2_TiO_3_

Li_2_TiO_3_ was eluted in 0.1 mol/L of HCl at 65 °C for 12 h, and then the eluate was changed again to ensure the elution. Finally, the eluted product was washed and filtered with ultrapure water and dried in an oven at 70 °C. The final product was spinel titanium oxide ion screen. The titanium-based lithium ion sieves H_2_TiO_3_ with high adsorption and specific adsorption for Li^+^ were obtained.

## 4. Adsorption Performance of H_2_TiO_3_

### 4.1. Effect of pH

**Figure 5:**
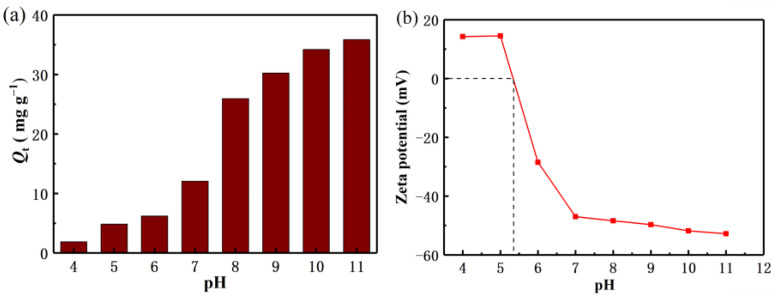
(**a**) Effect of pH on adsorption capacities (**b**) Zeta potential measurement of H_2_TiO_3_.

It is well known that the pH value of the solution plays an important role in the adsorption capacity of the adsorbent. In this paper, the relationship between the pH value and the adsorption mechanism of Li^+^ is briefly discussed. As shown in Figure 5a, with the increase of pH value, the adsorption capacity of Li^+^ also increases gradually. It can be seen that the adsorption of Li^+^ is favorable under alkaline conditions. In order to avoid adding more NaOH to regulate the higher pH concentration, thus increasing the role of cations in the solution, it is considered that the high pH value is not conducive to industrial mass production applications. Therefore, the recovery of Li^+^ in this experiment was mainly carried out at pH 11. Figure 5b shows the zeta potential test of the lithium-ion sieve. As the pH increases, the value of the electrostatic negative charge value on the material also gradually increases, consistent with the pH tests results.

### 4.2. Adsorption Kinetics

Through the adsorption kinetics experiment, the connection between the adsorption amount (*Q_t_*) and the adsorption time (*t*) was analyzed.

By setting different adsorption times, the residual Li^+^ concentration of the solution was tested. At a temperature of 298 K, the adsorption kinetics at different times (200, 400, 600, 800, 1000, 1200, 1400, 1600 min) was studied. The adsorption process of the ionic sieve H_2_TiO_3_ in LiCl solution was fitted by pseudo-first-order kinetic model (PFOKM) and pseudo-second-order kinetic model (PSOKM) to explore the adsorption rate constant and mechanism. The fitting equation of the PFOKM and PSOKM is as follows:

(1) $$Q_{t}=Q_{e}-e^{-k_{1}t}$$

(2) $$Q_{t}=\frac{k_{2}Q_{e}^{2}t}{1+k_{2}Q_{e}t}$$

where *Q_t_* (mg g^−1^) corresponds to the amount of Li^+^ adsorbed at time t (min), *Q_e_* (mg g^−1^) is the capacity of Li^+^ adsorbed when the adsorption process reaches equilibrium, and *t* (min) is the adsorption time. In addition, *k*_1_ and *k*_2_ are the rate constants of quasi first order and quasi second order dynamics, respectively.

Where h (mg g^−1^ min^−1^) represents the initial rate of the adsorption process, *t*_1/2_ (min) represents the semi-equilibrium time of adsorption. In addition, the h (mg g^−1^ min^−1^) and *t*_1/2_ (min) of the PSOKM are listed in the following Equations (3) and (4):

(3) $$h=k_{2}Q_{e}^{2}$$

(4) $$t_{1/2}=\frac{1}{k_{2}Q_{e}}$$

**Figure 6:**
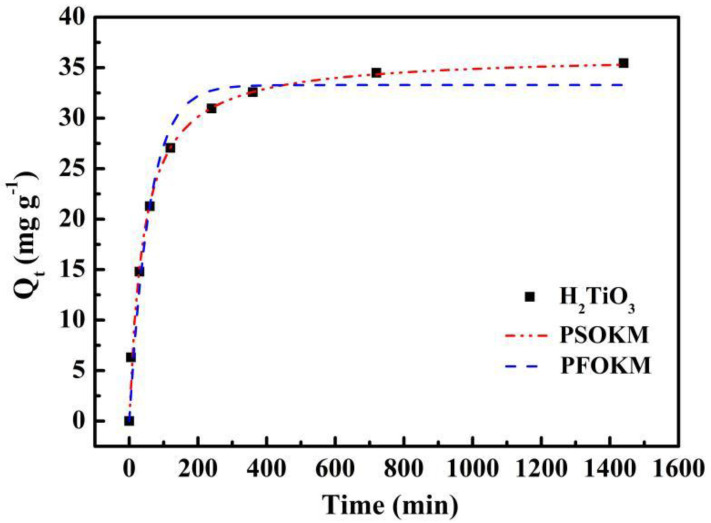
Kinetic data and modeling for the adsorption of Li^+^: Fitting curves of PFOKM and PSOKM.

The kinetic curve of adsorption was fitted by PFOKM and PSOKM. The fitting curve and corresponding parameters are presented in Figure 6 and Table 1, respectively. We can see in Figure 6 that the adsorption curve of the ion sieve adsorbent increases rapidly from the beginning, reaching about 80% of the maximum adsorption capacity at 200 min, then the adsorption curve increases slightly, and eventually gradually tends towards adsorption equilibrium at about 6 h.

Obviously, the correlation coefficient of *R*^2^ fitted by PSOKM is larger than the correlation coefficient of *R*^2^ fitted by PFOKM, which better matches the experimental data. At the same time, the equilibrium adsorption amount is calculated based on the PFOKM and the PSOKM. Compared with the actual values, the results of the pseudo-secondary dynamics model are closer to reality. Therefore, we believe that PSOKM is more in line with the adsorption of ion sieve H_2_TiO_3_ in LiCl solution. This further illustrates that the adsorption process of Li^+^ by H_2_TiO_3_ is mainly completed under the chemical action.

### 4.3. Adsorption Isotherms

**Figure 7:**
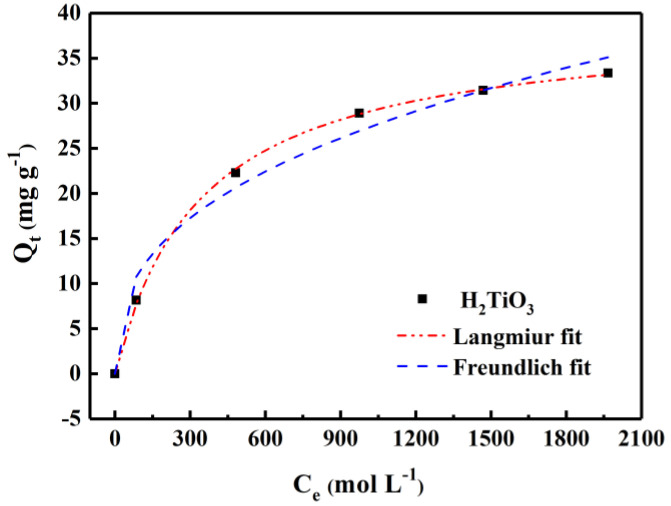
Isotherm model fitting of H_2_TiO_3_ adsorbing Li^+^.

By statically testing equilibrium adsorption data and adsorption curves in LiCl solutions (100–2000 mg L^−1^), we were able to explore the equilibrium concentration and adsorption amount of the adsorbent in different concentrations of lithium-containing solutions. Among them, from the curve fitted by the Langmuir and Freundlich equation (Figure 7), the adsorption effect will become higher with the increase of Li^+^ concentration. The saturation adsorption capacity of H_2_TiO_3_ is 35.45 mg g^−1^. The related isothermal constants are listed Table 2. It is not difficult to see that the correlation parameters of the Langmuir and Freundlich adsorption isotherm models are *R*^2^ = 0.996 and *R*^2^ = 0.978, respectively. In comparison, the Langmuir isotherm adsorption model can better match the experimental data, which also corresponds to the single layer adsorption. This indicates that fitting experimental data with the Langmuir equation is closer to the actual data. Hence, the lithium-ion sieve is known as an adsorbent with excellent performance in extracting Li^+^. The experimental data fit via the Langmuir and Freundlich models, which were calculated by Equations (5) and (6):

(5) $$Q_{e}=\frac{K_{L}Q_{m}C_{e}}{1+K_{L}C_{e}}$$

(6) $$Q_{e}=K_{F}C_{e}^{1/n}$$

Among them, *Q_m_* (mg g^−1^) is the maximum adsorption capacity of H_2_TiO_3_, and *K*_L_ (L g^−1^) is the affinity constant of the Langmuir isotherm, while the direction constant of the Freundlich isotherm is expressed by *K*_F_ (mg g^−1^). In addition, 1/n is called a heterogeneity factor. The *R*_L_ value indicates the separation factor, and the adsorption advantage of the adsorbent can be judged by the *R*_L_ value. The *R*_L_ value can be calculated by the following equation:

(7) $$R_{L}=\frac{1}{1+C_{m}K_{L}}$$

In this equation, *C_m_* is explained as the maximum initial concentration of Li^+^.

Figure 7 shows the adsorption isotherm of H_2_TiO_3_. It can be seen that the *R*^2^ value of the Langmuir adsorption model of H_2_TiO_3_ is 0.995, which is larger than that of the Freundlich adsorption model, indicating that the adsorption process of H_2_TiO_3_ is monomolecular chemical adsorption. When adsorption reached equilibrium, the adsorption capacity of H_2_TiO_3_ reached the maximum, which was 297 mg/g^−1^. In addition, if the *R*_L_ value is positive and less than 1, the smaller the *R*_L_ value, the more favorable it is for the adsorbent to adsorb Li^+^. Therefore, as can beseen from Table 1, the *R*_L_ value of H_2_TiO_3_ indicates that Li^+^ is easily captured by H_2_TiO_3_. Detailed data of adsorption isotherms are listed in Table 2.

### 4.4. Effect of Temperature

**Figure 8:**
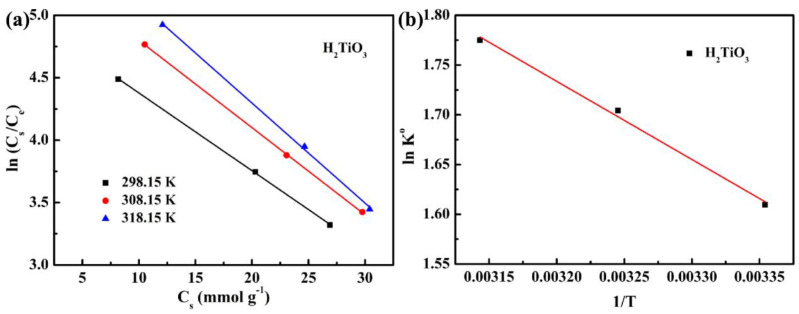
(**a**) the change of Q_e_ with temperature (**b**) the change of vant Hoff plot of lnK° to 1/T.

Further, we considered the influence of thermal motion characteristics on the adsorption efficiency. Therefore, we explored the effect of H_2_TiO_3_ on Li^+^ adsorption at different temperatures (298.15 K, 308.15 K, and 318.15 K) through thermodynamic experiments. ΔG° is calculated from the Gibbs free energy equation, and the change of *Q_e_* with temperature was studied (Figure 8a). ΔH° and ΔS° are calculated by the lnK° equation, and the change of vant Hoff plot of lnK° to 1/T was studied (Figure 8b) [31]. The results of the thermodynamic parameters of the ion sieve are shown in Table 3. It can be observed that at a temperature of 298.15–318.15 K, the value of ΔG° is maintained at −3.85 to −4.73 kJ mol^−1^, and is always negative. It indicates that as the temperature increases, the adsorption capacity will gradually become higher. That is to say, the adsorption of Li^+^ is a spontaneous process. At the same time, ΔH° is always positive, which can be explained as an endothermic adsorption process, indicating that the Li^+^ adsorption effect will increase with increasing temperature. Hence, we believe that the adsorption of Li^+^ on the ion sieve is a spontaneous endothermic process.

### 4.5. Selective and Reusability Tests

**Figure 9:**
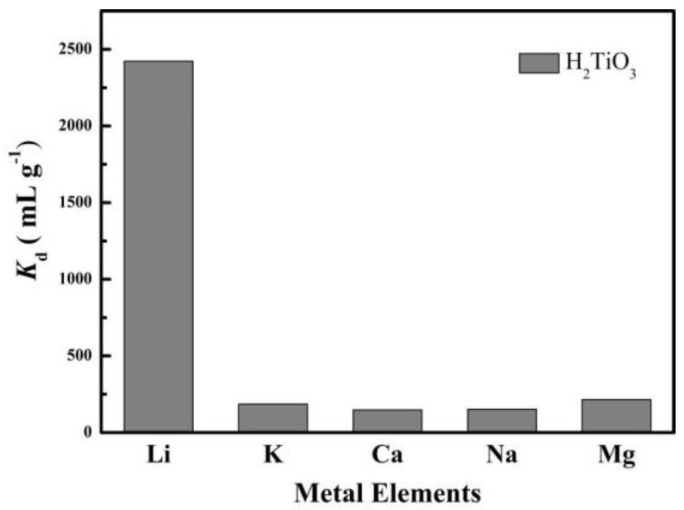
*K*_d_ values of H_2_TiO_3_ for a mixture of Li^+^, K^+^, Ca^2+^, Na^+^, Mg^2+^.

Through selective experiments to determine whether H_2_TiO_3_ has a specific adsorption selectivity for Li^+^, we simulated salt-lake brine for a competitive adsorption experiment, and tested the adsorption performance of H_2_TiO_3_ to Na^+^, Mg^2+^, K^+^, Ca^2+^, Li^+^. *K*_d_ (mL g^−1^) as the partition coefficient and *k* as the selectivity coefficient were used as indicators to evaluate the selectivity of H_2_TiO_3_. According to the magnitude of the comparison *K*_d_ value, the adsorption selection performance of H_2_TiO_3_ for Li^+^ can be judged. We have measured the *K*_d_ value of H_2_TiO_3_ for mixtures of Li^+^, K^+^, Ca^2+^, Na^+^, and Mg^2+^as shown in Figure 9. From the experimental results, we can clearly observe that when the adsorption reaches equilibrium, the adsorption effect and partition coefficient (*K*_d_) of Li^+^ by H_2_TiO_3_ are several times that of other metal ions. Based on this, it can be determined that the lithium ion sieve H_2_TiO_3_ specifically adsorbs Li^+^. The selectivity of the ion sieve for Li^+^ K^+^ Ca^2+^ Na^+^ Mg^2+^ was assessed by the distribution coefficient (*K*_d_, mL g^−1^), which is listed in Equation (7):

(8) $$K_{d}=\frac{C_{o}-C_{f}}{C_{f}}\times \frac{V}{m}$$

In this equation, *C*_0_ is interpreted as the initial concentration of five ionic solutions, and *C*_f_ represents the final concentration of each ionic solution. We also compared the *K*_d_ and *k* values of each ion pair H_2_TiO_3_ in Table 4, which shows that H_2_TiO_3_ has the largest *K*_d_ for lithium ions.

The industrial feasibility of the lithium ion sieve was verified by the adsorption cycle experiment, so that the production cost was minimized. After each adsorption, the eluent (0.1M HCl) desorbs the adsorbent. Reusability is one of the important characteristics used to evaluate the stability of the adsorbent. The regeneration experiment was repeated five times in total. Such a process is called a cycle. Desorb the adsorbed adsorbent H_2_TiO_3_ by HCl. After the desorption is completed, clean and dry, and repeat the next adsorption of Li^+^. The effect of H_2_TiO_3_ on the adsorption of Li^+^ was studied by five times of reweigh ability experiments.

**Figure 10:**
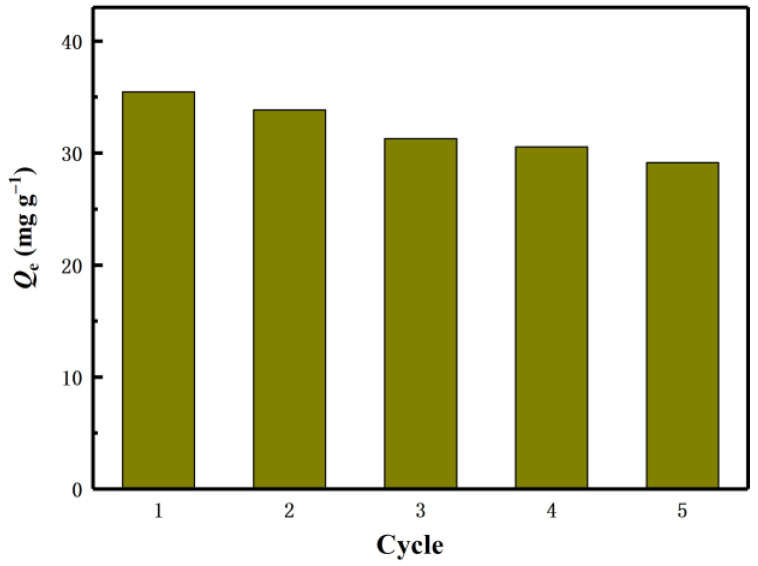
Regeneration of H_2_TiO_3_ five cycles.

The experimental results are shown in Figure 10. After five cycles, the capacity of the adsorbent remained above 82% of the initial value. All of these results confirm that H_2_TiO_3_ has a good cycle adsorption potential for Li^+^.

Here, we also compare work including this one with that already reported, showing the adsorption capacity of Li^+^ under similar conditions in Table 5.

## 5. Conclusions

To sum up, the characterization shows that we have successfully prepared a new lithium ion sieve with bacterial cellulose as the template, which has high specific surface area and can be separated by a simple and environmentally friendly low-temperature phase separation method. The results show that H_2_TiO_3_ is a single-layer chemical adsorption process, and has a good adsorption effect on lithium ions at pH 11.0, with a maximum adsorption capacity of 35.45 mg g^−1^, which is higher than the results of many previous studies. In addition, in the presence of other interfering metal ions, H_2_TiO_3_ still showed high selectivity for Li^+^, and the maximum adsorption capacity remained at 82% after five cycles of regeneration. To sum up, the ion sieve prepared by using cellulose as a biomimetic template in this study has the advantages of high adsorption capacity, good selectivity and strong regeneration ability, and is an ideal adsorbent for the efficient and selective adsorption of lithium. Therefore, we believe that the spinel-type titanium dioxide ion sieve prepared by this method is expected to have broad application prospects.

## Figures and Tables

**Table 1:** Kinetic parameters for the PFOKM and PSOKM.

Sorbents	*Q_e,_*_exp_ (mg g^−1^)	PFOKM			PSOKM		
		*Q_e_*_,c_ (mg g^−1^)	*k*_1_ (min^−1^)	*R* ^2^	*Q_e_*_,c_ (mg g^−1^)	*k*_2_ × 10^−2^ (g mg^−1^ min^−1^)	*R* ^2^
H_2_TiO_3_	35.45	33.28	0.0172	0.975	36.29	0.068	0.991

**Table 2:** Adsorption equilibrium constants of Langmuir and Freundlich models.

Sorbents	Langmuir Isotherm Model			Freundlich Isotherm Model		
	*Q_m_* (mg g^−1^)	*K*_L_ (L mg^−1^)	R^2^	*K*_F_ (mg g^−1^)	1/n	*R* ^2^
H_2_TiO_3_	38.96	0.003	0.998	2.01	0.38	0.948

**Table 3:** Thermodynamic parameters for Li^+^ adsorption.

Sorbents	∆H° (kJ mol^−1^)	∆S° (J mol^−1^)	T (*K*)	*K*°	∆G° (kJ mol^−1^)	*R* ^2^
H_2_TiO_3_	6.54	35.31	298.15	5.00	−3.99	0.992
			308.15	5.50	−4.37	
			318.15	5.90	−4.70	

**Table 4:** *K*_d_ and *k* values of H_2_TiO_3_.

Cation	H_2_TiO_3_		
	*C*_f_ (mg L^−1^)	*K*_d_ (mL g^−1^)	*k*
Li^+^	14.609	2422.548	
K^+^	48.059	40.388	0.017
Ca^2+^	45.961	87.879	2.176
Na^+^	47.634	49.670	0.565
Mg^2+^	44.087	134.121	2.700

**Table 5:** The adsorption capacity of Li^+^ under similar conditions.

Adsorbent	Adsorption Capacity	Reference Literature
Li_1.6_Mn_1.6_O_4_	44 mg g^−1^	(Gao et al. 2018) [3]
RHBC-Mnx	88.4mg g^−1^	(Yu et al. 2020) [29]
Li_2_CO_3_	32.6 mg g^−1^	(Chitrakar et al. 2014) [1]
β-Li_2_TiO_3_	30.4mg g^−1^	(Wang et al. 2016) [22]
H_2_TiO_3_	35.45 mg g^−1^	

## Data Availability

The authors confirm that the data supporting the findings of this study are available within the article.
